# Examining the Effect of Genes on Depression as Mediated by Smoking and Modified by Sex

**DOI:** 10.3390/genes15050565

**Published:** 2024-04-27

**Authors:** Kirsten Voorhies, Julian Hecker, Sanghun Lee, Georg Hahn, Dmitry Prokopenko, Merry-Lynn McDonald, Alexander C. Wu, Ann Wu, John E. Hokanson, Michael H. Cho, Christoph Lange, Karin F. Hoth, Sharon M. Lutz

**Affiliations:** 1 Department of Population Medicine, Harvard Pilgrim Health Care Institute, Boston, MA 02215, USA; 2 Channing Division of Network Medicine, Brigham and Women’s Hospital and Harvard Medical School, Boston, MA 02115, USA; 3 Division of Medicine, Department of Medical Consilience, Graduate School, Dankook University, Yongin 16890, Republic of Korea; 4 Brigham and Women’s Hospital, Division of Pharmacoepidemiology and Pharmacoeconomics, and Department of Medicine, Harvard Medical School, Boston, MA 02120, USA; 5 Genetics and Aging Research Unit and the McCance Center for Brain Health, Department of Neurology, Massachusetts General Hospital, Boston, MA 02114, USA; 6 Department of Epidemiology, School of Public Health, University of Alabama at Birmingham, Birmingham, AL 35233, USA; 7 Division of Pulmonary, Allergy and Critical Care Medicine, Department of Medicine, University of Alabama at Birmingham, Birmingham, AL 35294, USA; 8 Department of Genetics, University of Alabama at Birmingham, Birmingham, AL 35233, USA; 9 Harvard College, Cambridge, MA 02138, USA; 10 Department of Epidemiology, University of Colorado Anschutz Medical Campus, Aurora, CO 80045, USA; 11 Department of Biostatistics, T.H. Chan School of Public Health, Harvard University, Boston, MA 02115, USA; 12 Department of Psychiatry and Iowa Neuroscience Institute, University of Iowa, Iowa City, IA 52242, USA

**Keywords:** depression, mediation analysis, smoking, sex moderation

## Abstract

Depression is heritable, differs by sex, and has environmental risk factors such as cigarette smoking. However, the effect of single nucleotide polymorphisms (SNPs) on depression through cigarette smoking and the role of sex is unclear. In order to examine the association of SNPs with depression and smoking in the UK Biobank with replication in the COPDGene study, we used counterfactual-based mediation analysis to test the indirect or mediated effect of SNPs on broad depression through the log of pack-years of cigarette smoking, adjusting for age, sex, current smoking status, and genetic ancestry (via principal components). In secondary analyses, we adjusted for age, sex, current smoking status, genetic ancestry (via principal components), income, education, and living status (urban vs. rural). In addition, we examined sex-stratified mediation models and sex-moderated mediation models. For both analyses, we adjusted for age, current smoking status, and genetic ancestry (via principal components). In the UK Biobank, rs6424532 [*LOC105378800*] had a statistically significant indirect effect on broad depression through the log of pack-years of cigarette smoking (*p* = 4.0 × 10^−4^) among all participants and a marginally significant indirect effect among females (*p* = 0.02) and males (*p* = 4.0 × 10^−3^). Moreover, rs10501696 [*GRM5*] had a marginally significant indirect effect on broad depression through the log of pack-years of cigarette smoking (*p* = 0.01) among all participants and a significant indirect effect among females (*p* = 2.2 × 10^−3^). In the secondary analyses, the sex-moderated indirect effect was marginally significant for rs10501696 [*GRM5*] on broad depression through the log of pack-years of cigarette smoking (*p* = 0.01). In the COPDGene study, the effect of an SNP (rs10501696) in *GRM5* on depressive symptoms and medication was mediated by log of pack-years (*p* = 0.02); however, no SNPs had a sex-moderated mediated effect on depressive symptoms. In the UK Biobank, we found SNPs in two genes [*LOC105378800*, *GRM5*] with an indirect effect on broad depression through the log of pack-years of cigarette smoking. In addition, the indirect effect for *GRM5* on broad depression through smoking may be moderated by sex. These results suggest that genetic regions associated with broad depression may be mediated by cigarette smoking and this relationship may be moderated by sex.

## 1. Introduction

Depression is extremely common, affecting over 300 million people worldwide [1]. Depression has both genetic [2,3] and environmental influences, such as cigarette smoking [4,5]. In addition, women are more likely to be affected by depression than men [6,7]. However, the relationship between genes and depression is complex and the role of sex and cigarette smoking is unclear.

Genome-wide association studies (GWASs) have provided evidence that major depression is a polygenic trait with over 100 loci associated with depression [8,9,10,11,12]. Sex-stratified GWASs of depression have found novel variants. For example, a GWAS of major depressive disorder stratified by sex in two large biobanks, Generation Scotland and UK Biobank, found that the genes *CRTAP*, *GLB1*, and *TMPPE* at chromosome 3p22.3 were significantly associated with major depressive disorder in males, but not in females [13]. Linkage disequilibrium (LD) score regression analyses of GWASs have found statistically significant genetic correlations between smoking initiation, cigarettes smoked per day (CPD), and depressive symptoms [14]. Despite there being a genetic association between smoking and depression [12,15], few studies have examined the role of smoking and sex on genes associated with depression. A recent study examining sex differences in pleiotropic effects for depression and smoking found pleiotropic effects of *FKBP5* on depression and smoking initiation among all participants and pleiotropy for *NR3C2* and *CHRNA5* for depression and cigarettes per day among females [16].

A recent GWAS in the UK Biobank found 14 single nucleotide polymorphisms (SNPs) associated with broad depression, where broad depression was defined as having seen a doctor or psychiatrist for “nerves, anxiety, tension or depression” or having had a depressive mood disorder diagnosis [17]. While nine of these SNPs have previously been associated with cigarette smoking or incorporated in smoking cessation studies, the role of sex and smoking on the association with these SNPs and broad depression was not explored. Here, we first conducted a mediation analysis to estimate the indirect effect of these 14 SNPs on broad depression through the mediator, the log of pack-years of cigarette smoking, in the UK Biobank, our primary study population [18,19,20,21,22,23,24]. The COPDGene study served as a replication sample with the advantage of including individuals enriched for a history of cigarette smoking exposure (i.e., individuals with ≥10 pack-years were enrolled). In the COPDGene study, we examined the effect of these 14 SNPs on depressive symptoms through the mediator, the log of pack-years of cigarette smoking. Finally, in addition to the mediation analysis in the two cohorts, we also used a moderated mediation analysis, also known as conditional indirect effects, to examine if the indirect effect of the SNPs on depression through the log of pack-years of cigarette smoking differs by the moderator, sex [25,26,27,28].

## 2. Materials and Methods

### 2.1. Primary Population: UK Biobank

The UK Biobank is a large prospective study that recruited over 500,000 participants in the United Kingdom. Biological and medical data were collected, and genotypic data are available for about 488,000 participants [29]. We excluded participants of European ancestry with zero pack-years of cigarette smoking and related individuals using kinship coefficients. Broad depression was defined as a positive response to one of the following questions: “Seen doctor (GP) for nerves, anxiety, tension or depression” or “Seen a psychiatrist for nerves, anxiety, tension or depression”, or a depressive mood disorder diagnosis (defined as a primary or secondary diagnosis from linked hospital admission records) [17]. Similar to Howard et al. [17] using primary and secondary diagnoses, we excluded individuals with ICD codes for the following: bipolar, multiple personality disorder, schizophrenia/psychosis, and controls with ICD codes for mood disorders. Additionally, using treatment/medication codes, we excluded participants with codes for antipsychotics and controls with codes for antidepressants. This resulted in 97,330 participants for the analysis as summarized in Table 1.

### 2.2. Replication Population: COPDGene

The COPDGene study, a multicenter observational study, recruited 10,192 adults with a history of smoking exposure (current and former smoker) with at least 10 pack-years of smoking history who were non-Hispanic whites or African Americans. The COPDGene study was designed to identify genetic factors associated with COPD [30]. For our analyses, we restricted the analysis to participants of European ancestry with available phenotypic information (N = 3829). In the COPDGene study, we defined cases based on the Hospital Anxiety and Depression Scale depression subscale score (HADS-D ≥ 8) and/or the use of an antidepressant medication [31]. Participants with no HADS-D score or a missing depression medication variable were excluded.

### 2.3. Statistical Analyses

Of the 14 SNPs associated with broad depression from the GWAS by Howard et al. [17], 9 SNPs were previously associated with smoking or incorporated in smoking cessation studies [32,33,34,35,36,37,38,39,40,41,42,43]. Given the potential pleiotropic relationship between these SNPs with broad depression and cigarette smoking, we examined the indirect effect of the 14 SNPs on depression through the mediator, natural log of pack-years of cigarette smoking, using a counterfactual-based mediation analysis implemented in the R package “mediation” in the UK Biobank and the COPDGene study [22]. In the primary analysis, we adjusted for sex, age, current smoking status, and genetic ancestry using the first 8 principal components (PCs) in the UK Biobank, similar to Howard et al. [17]. In an additional analysis, we adjusted for the covariates above plus income, level of education, and rural vs. urban location. Income was defined as a low income in the UK Biobank if the income was less than £18,000, not a low income if the income was above £18,000, and participants that stated they did not know their income or they preferred not to answer were a separate income category. Low income in the COPDGene study was defined as an income less than $15,000, not low income was defined as an income greater than $15,000, and participants that declined to answer were a separate income category. Education in the UK Biobank and the COPDGene study was defined as college degree or higher versus all other categories. Urban location was defined in the UK Biobank as a population ≥ 10,000. In the COPDGene study, urban location was defined as a metropolitan or micropolitan area. In the secondary analyses, we stratified by sex and examined if sex moderated the indirect effect of the SNPs on broad depression through the mediator, natural log of pack-years of cigarette smoking, using moderated mediation analysis. For the sex-stratified and sex-moderated mediation analyses, we adjusted for the primary set of covariates (age, current smoking status, and genetic ancestry using the first 8 PCs) and, in an additional analysis, we adjusted for the primary set of covariates plus income, education, and rural vs. urban location. We repeated all analyses in the COPDGene study for an outcome based on depressive symptoms and medication use.

## 3. Results

### 3.1. Characteristics of Participants

The characteristics of participants included in the analyses from the UK Biobank and COPDGene are shown in Table 1. We included 97,330 participants who were current or former smokers of European ancestry from the UK Biobank and 3829 current or former smokers of European ancestry from the COPDGene study. The mean age of participants was 57.7 and 67.7 years in the UK Biobank and COPDGene study, respectively. For the outcome, 40.1% and 33.2% of participants were classified as cases for broad depression in the UK Biobank and depressive symptoms and medication use in the COPDGene study, respectively. The majority of participants in both cohorts were male and former smokers. The mean pack-years of smoking was greater for the COPDGene cohort as compared to the UK Biobank cohort (46.3 vs. 23.9).

### 3.2. Mediation Analysis

Using Bonferroni correction, we defined the significance threshold as 0.05/14 = 3.6 × 10^−3^. The results of the mediation and moderated mediation analyses are given in Table 2 adjusting for age, sex, current smoking status, and genetic ancestry via PCs. Among all participants in the UK Biobank, one SNP (rs6424532 [*LOC105378800*]) had a significant indirect effect on broad depression through log of pack-years (*p* = 4.0 × 10^−4^) and one SNP (rs10501696 [*GRM5*]) had a marginally significant indirect effect (*p* = 0.01). In the COPDGene study, the effect of rs180838672 [*GRM5*] on depressive symptoms was mediated by log of pack-years (*p* = 0.02) as seen in Table 3. Note that the SNP in the UK Biobank (rs10501696) was not available in the COPDGene study; so another SNP rs180838672 [*GRM5*] was used for the analyses. In Supplementary Tables S1 and S2, we adjusted for age, sex, current smoking status, genetic ancestry via PCs, education, income, and location (urban vs. rural). Similar results were obtained for both sets of covariate adjustments.

### 3.3. Sex-Stratified and Sex-Moderated Mediation Analyses

In the sex-stratified analysis, one SNP (rs10501696 [*GRM5*]) had a significant indirect effect on broad depression through log of pack-years among females (*p* = 2.2 × 10^−3^) and two SNPs (rs6424532 [*LOC105378800*], and rs263575 [*BNC2*/*CNTLN*]) had a marginally significant indirect effect among females (*p* = 0.02 and *p* = 0.03, respectively). Two SNPs (rs6424532 [*LOC105378800*], and rs2402273 [*LSM8*/*CTTNBP2*]) had a marginally significant indirect effect on broad depression through log of pack-years among males (*p* = 4.0 × 10^−3^ and *p* = 4.1 × 10^−3^, respectively). One SNP (rs10501696 [*GRM5*]) had a marginally significant sex-moderated indirect effect on broad depression through log of pack-years (*p* = 0.01). In the COPDGene study, no SNP had a sex-moderated mediated effect on depressive symptoms.

## 4. Discussion

The findings from the current study in the UK Biobank provide evidence that SNPs associated with broad depression may be mediated by smoking and moderated by sex. First, we found one SNP (rs6424532 [*LOC105378800*]) that had a significant indirect effect and one SNP (rs10501696 [*GRM5*]) that had a marginally significant indirect effect on broad depression through smoking (defined by the log of pack-years of cigarette smoking) among participants of European ancestry in the UK Biobank. While rs6424532 [*LOC105378800*] had a significant indirect effect on broad depression through the log of pack-years of cigarette smoking, this SNP had a marginally significant indirect effect on broad depression among both males and females. Note that while rs10510696 [*GRM5*] had a marginally significant indirect effect on broad depression through the log of pack-years of cigarette smoking, this SNP had a significant indirect effect on broad depression among females but not males and had a marginally significant sex-moderated effect on broad depression. The indirect effects of the SNPs rs6424532 [*LOC105378800*] and rs10510696 [*GRM5*] on broad depression through pack-years of cigarette smoking for all participants, males, and females in the UK Biobank are displayed in Supplementary Figures S1 and S2. As seen in the plots, the indirect effect of rs10510696 [*GRM5*] on broad depression is significant among females but not males. As seen in Supplemental Figures S3 and S4, there appears to be a stronger sex by SNP by pack-years interaction on broad depression for rs10510696 [*GRM5*] than rs6424532 [*LOC105378800*].

In addition, rs263575 [*BNC2*/*CNTLN*] had a marginally significant indirect effect on broad depression among females but not males, and rs2402273 [*LSM8*/*CTTNBP2*] had a marginally significant indirect effect on broad depression among males but not females. In the COPDGene study, the effect of rs180838672 [*GRM5*] on depressive symptoms was mediated by log of pack-years (*p* = 0.02) among all subjects of European ancestry. Note that the SNP in the UK Biobank (rs10501696) was not available in the COPDGene study so another adjacent SNP rs180838672 [*GRM5*] was used for the analyses. Also, note that the outcome in the UK Biobank was broad depression and the outcome in the COPDGene study was depressive symptoms. Note that in our analyses that adjusted for additional covariates (urban location, income, and education), the results were similar, as seen in Supplemental Tables S1 and S2.

While *LOC105378800* was previously associated with broad depression, this gene does not appear to have been previously associated with smoking behavior. *GRM5* (Glutamate Metabotropic Receptor 5) was previously associated with smoking behavior [42] and incorporated in smoking cessation studies [32,43] and has also previously been associated with depression [44,45,46]. Both *LSM8*/*CTTNBP2* and *BNC2*/*CNTLN* were incorporated in smoking cessation studies [32,33]. In addition, *LSM8*/*CTTNBP2* was previously associated with lifetime smoking [34], smoking initiation [35,36,37], and smoking status [38,39,40,41].

The strengths of this study include the fact that the primary analysis was conducted in a large, prospective biobank with well-described phenotypes that include broad depression and the log of pack-years of cigarette smoking. Nevertheless, there are several limitations of this study. While multiple regions had previously been incorporated in smoking cessation studies, it is not clear how much of a role smoking played in these previous findings. Additionally, the UK Biobank does not have a full interview specific to current and past history of major depression. Furthermore, while we used the definition of broad depression based on the UK Biobank GWAS of broad depression [17] defined as having seen a doctor or psychiatrist for “nerves, anxiety, tension or depression” or having had a depressive mood disorder diagnosis, this definition of broad depression may also include anxiety disorders, neurasthenia, somatoform disorders, etc., or even isolated symptoms of major depressive disorder (MDD). By applying this definition, the heterogeneity of the study group is increased versus patients who received a diagnosis of a certain depressive disorder from a psychiatrist. This heterogeneity may negatively impact the results of the current study. Also, note that excluding patients receiving antipsychotics in the UK Biobank does not necessarily mean that only patients with psychotic disorders were eliminated from the study group, since patients with major depression may receive such pharmacological agents as add-ons for severe cases or for the treatment of MDD with psychotic features. For example, smoking is an inducer of the *CYP1A2* isoenzyme; therefore, in severe cases of nicotine use disorder, adding other agents to the antidepressant may be required due to the lower plasma concentrations of pharmacological agents. In addition, the mediator was based on pack-years of cigarette smoking excluding participants with zero pack-years of smoking history and never smokers. Occasional smoking and nicotine use disorder cannot be put on the same level when exploring the association of such a variable with other clinical or demographic variables. Non-zero pack-years of smoking may be an insufficient characterization of the study group.

Note that some of these SNPs were previously cited in a smoking cessation trial, but the specific role of these SNPs in this smoking cessation study is not clear [32]. For the smoking cessation manuscript, genotype score of quit success for quitting smoking was created. For the genotype scores, alleles at 12,058 SNPs were examined. For these, one or more of three clinical trials of smoking cessation success found them to be different in quitters who were successful versus not, at significance level of *p* < 0.01. While some of the 14 SNPs were included in this genotype score, it is not clear how much they were individually associated with smoking cessation.

In conclusion, we examined the effect of 14 SNPs previously associated with broad depression through the log of pack-years of cigarette smoking and we found two regions [*LOC105378800*, *GRM5*] where the association with broad depression was mediated by smoking. For *GRM5*, the indirect effect of the SNP in this region on broad depression through the log of pack-years of cigarette smoking in the UK Biobank may be moderated by sex.

For future directions, it would be important to investigate the mechanisms by which the identified SNPs influence depression through smoking. More studies are needed to examine the effect of these SNPs on depression through smoking as modified by sex.

## Figures and Tables

**Table 1:** Characteristics of participants from the UK Biobank and COPDGene.

	UK Biobank	COPDGene
Sample size, N	97,330	3829
Depression, N [%]	38,999 [40.1%]	1270 [33.2%]
Sex (male), N [%]	51,292 [52.7%]	1941 [50.7%]
Age, mean [SD]	57.7 [7.8]	67.7 [8.3]
Current Smoker, N [%]	25,869 [26.6%]	1050 [27.4%]
Pack-years, mean [SD]	23.9 [18.6]	46.3 [24.7]
Location: Urban, N [%]	83,188 [86.6%]	3613 [94.4%]
Income: Low, N [%]	22,888 [23.8%]	639 [16.7%]
Income: Not low, N [%]	61,166 [63.7%]	2721 [71.1%]
Income: Not disclosed, N [%]	11,966 [12.5%]	468 [12.2%]
Education: College degree or greater, N [%]	23,125 [24.1%]	1814 [47.4%]

**Table 2:** Mediation, sex-stratified mediation, and sex-moderated mediation results for the indirect effect of the SNP on depression through log of pack-years of cigarette smoking in the UK Biobank adjusting for age, sex, current smoking status, and genetic ancestry via PCs. There were 46,038 female participants and 51,292 male participants included.

Chr	Marker	Gene/Nearest Gene	Position	Allele Freq.	Prev. Smok. Assoc. **	Indirect Effect (All)			Indirect Effect (Female)			Indirect Effect (Male)			Sex-Moderated Indirect Effect		
						β	95% CI	*p*	β	95% CI	*p*	β	95% CI	*p*	β	95% CI	*p*
1	rs10127497	*SGIP1*	66584461	0.14	32	−8.5 × 10^−6^	(−4.2 × 10^−4^, 4.1 × 10^−4^)	0.99	−2.4 × 10^−5^	(−7.2 × 10^−4^, 6.7 × 10^−4^)	0.94	−3.0 × 10^−6^	(−4.9 × 10^−4^, 5.0 × 10^−4^)	0.98	−1.1 × 10^−6^	(−8.3 × 10^−4^, 6.9 × 10^−4^)	0.87
1	rs6699744	*LOC105378797*	72359461	0.61	-	1.7 × 10^−4^	(−1.3 × 10^−4^, 4.5 × 10^−4^)	0.27	2.1 × 10^−4^	(−2.8 × 10^−4^, 7.3 × 10^−4^)	0.43	1.3 × 10^−4^	(−1.9 × 10^−4^, 4.8 × 10^−4^)	0.42	−1.9 × 10^−5^	(−6.1 × 10^−4^, 6.3 × 10^−4^)	0.94
1	rs6424532	*LOC105378800*	73198339	0.49	-	4.9 × 10^−4^	(2.1 × 10^−4^, 7.7 × 10^−4^)	4.0 × 10^−4^	5.7 × 10^−4^	(1.1 × 10^−4^, 1.1 × 10^−3^)	0.02	4.1 × 10^−4^	(1.1 × 10^−4^, 7.4 × 10^−4^)	4.0 × 10^−3^	−1.1 × 10^−4^	(−6.3 × 10^−4^, 4.6 × 10^−4^)	0.76
1	rs7548151	*ASTN1*	177057847	0.08	32, 33	3.7 × 10^−4^	(−1.6 × 10^−4^, 8.8 × 10^−4^)	0.17	1.4 × 10^−4^	(−7.5 × 10^−4^, 1.1 × 10^−3^)	0.79	5.1 × 10^−4^	(−7.6 × 10^−5^, 1.1 × 10^−3^)	0.11	3.9 × 10^−4^	(−5.5 × 10^−4^, 1.3 × 10^−3^)	0.51
5	rs40465	*LOC105379109*	104646025	0.33	-	−6.5 × 10^−5^	(−3.6 × 10^−4^, 2.4 × 10^−4^)	0.69	−8.2 × 10^−5^	(−5.7 × 10^−4^, 4.7 × 10^−4^)	0.83	−5.5 × 10^−5^	(−3.9 × 10^−4^, 3.0 × 10^−4^)	0.72	4.6 × 10^−5^	(−6.6 × 10^−4^, 7.0 × 10^−4^)	0.88
6	rs3132685	*HCG9*	29978172	0.13	-	3.7 × 10^−4^	(−4.3 × 10^−5^, 7.9 × 10^−4^)	0.08	5.6 × 10^−4^	(−1.5 × 10^−4^, 1.4 × 10^−3^)	0.12	2.1 × 10^−4^	(−2.6 × 10^−4^, 6.7 × 10^−4^)	0.41	−3.5 × 10^−4^	(−1.3 × 10^−3^, 5.3 × 10^−4^)	0.38
6	rs112348907	*KCNQ5*	72878230	0.30	32	−2.4 × 10^−5^	(−3.3 × 10^−4^, 2.9 × 10^−4^)	0.90	6.3 × 10^−5^	(−4.5 × 10^−4^, 6.2 × 10^−4^)	0.78	−6.8 × 10^−5^	(−4.2 × 10^−4^, 2.6 × 10^−4^)	0.67	−1.3 × 10^−4^	(−8.0 × 10^−4^, 4.9 × 10^−4^)	0.78
7	rs3807865	*TMEM106B*	12210776	0.41	-	1.9 × 10^−4^	(−9.9 × 10^−5^, 4.6 × 10^−4^)	0.18	4.2 × 10^−4^	(−9.4 × 10^−5^, 9.2 × 10^−4^)	0.10	4.8 × 10^−5^	(−2.7 × 10^−4^, 3.6 × 10^−4^)	0.78	−3.7 × 10^−4^	(−8.9 × 10^−4^, 1.5 × 10^−4^)	0.26
7	rs2402273	*LSM8/* *CTTNBP2*	117960370	0.41	32, 34–41	2.5 × 10^−4^	(−7.5 × 10^−6^, 5.3 × 10^−4^)	0.06	−6.1 × 10^−5^	(−5.6 × 10^−4^, 4.5 × 10^−4^)	0.81	4.4 × 10^−4^	(1.2 × 10^−4^, 7.6 × 10^−4^)	4.1 × 10^−3^	5.2 × 10^−4^	(4.2 × 10^−5^, 1.1 × 10^−3^)	0.05
9	rs263575	*BNC2*/*CNTLN*	17033842	0.46	32, 33	−2.4 × 10^−4^	(−5.3 × 10^−4^, 3.1 × 10^−5^)	0.08	−5.6 × 10^−4^	(−1.1 × 10^−3^,−6.2 × 10^−5^)	0.03	−3.7 × 10^−5^	(−3.6 × 10^−4^, 3.1 × 10^−4^)	0.82	5.1 × 10^−4^	(5.3 × 10^−6^, 1.1 × 10^−3^)	0.05
10	rs1021363	*SORCS3*	104851081	0.64	32	−2.8 × 10^−4^	(−5.8 × 10^−4^, 8.9 × 10^−6^)	0.06	−3.9 × 10^−4^	(−8.7 × 10^−4^, 1.7 × 10^−4^)	0.15	−2.1 × 10^−4^	(−5.3 × 10^−4^, 1.2 × 10^−4^)	0.22	2.1 × 10^−4^	(−4.2 × 10^−4^, 9.1 × 10^−4^)	0.45
11	rs10501696	*GRM5*	89014994	0.50	32, 42, 43	−3.7 × 10^−4^	(−6.7 × 10^−4^, −7.2 × 10^−5^)	0.01	−8.0 × 10^−4^	(−1.3 × 10^−3^, −2.9 × 10^−4^)	2.2 × 10^−3^	−8.6 × 10^−5^	(−4.2 × 10^−4^, 2.4 × 10^−4^)	0.57	7.1 × 10^−4^	(1.7 × 10^−4^, 1.3 × 10^−3^)	0.01
13	rs9530139	*B3GLCT*	31273187	0.19	32	−7.3 × 10^−5^	(−4.1 × 10^−4^, 3.0 × 10^−4^)	0.71	−3.1 × 10^−4^	(−9.6 × 10^−4^, 2.6 × 10^−4^)	0.32	7.4 × 10^−5^	(−3.1 × 10^−4^, 5.0 × 10^−4^)	0.73	4.0 × 10^−4^	(−3.2 × 10^−4^, 1.2 × 10^−3^)	0.30
15	rs28541419	*MRPL46*	88402647	0.23	32	1.4 × 10^−4^	(−2.0 × 10^−4^, 4.9 × 10^−4^)	0.35	−5.8 × 10^−5^	(−6.3 × 10^−4^, 5.3 × 10^−4^)	0.86	2.5 × 10^−4^	(−9.0 × 10^−5^, 6.4 × 10^−4^)	0.17	2.9 × 10^−4^	(−4.1 × 10^−4^, 9.5 × 10^−4^)	0.46

Note: cells are highlighted green when *p* < 0.05/14, and cells are highlighted yellow when 0.05/14 ≤ *p* < 0.05. ** Full citations listed in References section.

**Table 3:** Mediation, sex-stratified mediation, and sex-moderated mediation results for the indirect effect of the SNP on depressive symptoms through log of pack-years of cigarette smoking in the COPDGene study adjusting for age, sex, current smoking status, and genetic ancestry via PCs.

Chr	Marker	Gene/Nearest Gene	Position	Allele Freq.	Prev. Smok. Assoc. **	Indirect Effect (All)			Indirect Effect (Female)			Indirect Effect (Male)			Sex-Moderated Indirect Effect		
						β	95% CI	*p*	β	95% CI	*p*	β	95% CI	*p*	β	95% CI	*p*
1	rs10127497	*SGIP1*	66584461	0.14	32	−1.0 × 10^−4^	(−1.8 × 10^−3^, 1.6 × 10^−3^)	0.89	−7.2 × 10^−4^	(−4.4 × 10^−3^, 2.4 × 10^−3^)	0.64	1.9 × 10^−4^	(−1.4 × 10^−3^, 2.0 × 10^−3^)	0.77	3.1 × 10^−4^	(−2.2 × 10^−3^, 2.9 × 10^−3^)	0.87
1	rs12143898 *	*LOC105378797*	72360489 *	0.20	-	2.3 × 10^−4^	(−1.2 × 10^−3^, 1.6 × 10^−3^)	0.73	−8.3 × 10^−4^	(−4.0 × 10^−3^, 1.7 × 10^−3^)	0.58	5.6 × 10^−4^	(−6.7 × 10^−4^, 2.8 × 10^−3^)	0.44	1.5 × 10^−3^	(−5.3 × 10^−4^, 4.6 × 10^−3^)	0.19
1	rs12044445 *	*LOC105378800*	73200931 *	0.47	-	8.1 × 10^−5^	(−1.1 × 10^−3^, 1.4 × 10^−3^)	0.84	−8.9 × 10^−4^	(−3.6 × 10^−3^, 1.2 × 10^−3^)	0.41	4.3 × 10^−4^	(−5.4 × 10^−4^, 2.0 × 10^−3^)	0.45	8.6 × 10^−4^	(−7.0 × 10^−4^, 3.0 × 10^−3^)	0.31
1	rs7548151	*ASTN1*	177057847	0.09	32, 33	−4.2 × 10^−4^	(−2.6 × 10^−3^, 1.7 × 10^−3^)	0.70	−8.3 × 10^−4^	(−5.6 × 10^−3^, 3.6 × 10^−3^)	0.71	−2.4 × 10^−4^	(−2.7 × 10^−3^, 1.7 × 10^−3^)	0.82	4.3 × 10^−4^	(−3.4 × 10^−3^, 3.8 × 10^−3^)	0.91
5	rs40465	*LOC105379109*	104646025	0.33	-	1.1 × 10^−3^	(3.8 × 10^−5^, 2.6 × 10^−3^)	0.05	3.0 × 10^−3^	(5.3 × 10^−4^, 6.6 × 10^−3)^	0.02	1.4 × 10^−4^	(−1.0 × 10^−3^, 1.5 × 10^−3^)	0.76	−1.6 × 10^−3^	(−4.4 × 10^−3^, 6.1 × 10^−4^)	0.18
6	rs112348907	*KCNQ5*	72878230	0.29	-	1.2 × 10^−3^	(−6.4 × 10^−5^, 3.1 × 10^−3^)	0.07	7.4 × 10^−4^	(−1.7 × 10^−3^, 3.4 × 10^−3^)	0.51	1.3 × 10^−3^	(−4.6 × 10^−4^, 4.2 × 10^−3^)	0.18	6.9 × 10^−4^	(−1.6 × 10^−3^, 3.2 × 10^−3^)	0.46
7	rs3807865	*TMEM106B*	12210776	0.41	32	−4.5 × 10^−4^	(−1.7 × 10^−3^, 6.5 × 10^−4^)	0.44	4.8 × 10^−4^	(−1.9 × 10^−3^, 3.1 × 10^−3^)	0.68	−7.1 × 10^−4^	(−2.7 × 10^−3^, 4.2 × 10^−4^)	0.28	−1.1 × 10^−3^	(−3.7 × 10^−3^, 9.0 × 10^−4^)	0.30
7	rs2402273	*LSM8/* *CTTNBP2*	117960370	0.42	-	6.9 × 10^−4^	(−4.0 × 10^−4^, 2.0 × 10^−3^)	0.27	4.5 × 10^−4^	(−2.1 × 10^−3^, 2.9 × 10^−3^)	0.69	5.5 × 10^−4^	(−4.5 × 10^−4^, 2.3 × 10^−3^)	0.37	3.1 × 10^−4^	(−2.5 × 10^−3^, 3.1 × 10^−3^)	0.81
9	rs263575	*BNC2*/*CNTLN*	17033842	0.45	32, 34–41	−1.8 × 10^−4^	(−1.4 × 10^−3^, 1.0 × 10^−3^)	0.79	4.1 × 10^−4^	(−1.9 × 10^−3^, 2.8 × 10^−3^)	0.75	−3.5 × 10^−4^	(−2.2 × 10^−3^, 6.7 × 10^−4^)	0.58	−5.0 × 10^−4^	(−2.3 × 10^−3^, 1.1 × 10^−3^)	0.64
10	rs79699572 *	*SORCS3*	105109590 *	0.03	32, 33	−3.1 × 10^−3^	(−6.9 × 10^−3^, −4.1 × 10^−5^)	0.04	−4.7 × 10^−3^	(−0.01, 9.4 × 10^−4^)	0.12	−1.7 × 10^−3^	(−6.7 × 10^−3^, 1.4 × 10^−3^)	0.36	7.8 × 10^−4^	(−5.3 × 10^−3^, 6.9 × 10^−3^)	0.77
11	rs180838672 *	*GRM5*	88584239 *	0.01	32	−9.0 × 10^−3^	(−0.02, −1.1 × 10^−3^)	0.02	−0.02	(−0.04, 2.2 × 10^−3^)	0.10	−4.2 × 10^−3^	(−0.01, 2.4 × 10^−3^)	0.24	8.4 × 10^−3^	(−7.4 × 10^−3^, 0.03)	0.40
13	rs9530139	*B3GLCT*	31273187	0.19	32, 42, 43	−1.1 × 10^−3^	(−3.0 × 10^−3^, 3.4 × 10^−4^)	0.15	−1.4 × 10^−3^	(−4.6 × 10^−3^, 1.6 × 10^−3^)	0.36	−8.1 × 10^−4^	(−3.1 × 10^−3^, 5.5 × 10^−4^)	0.33	−3.8 × 10^−6^	(−2.8 × 10^−3^, 3.3 × 10^−3^)	0.92
15	rs28541419	*MRPL46*	88402647	0.24	32	−1.1 × 10^−3^	(−2.9 × 10^−3^, 2.9 × 10^−4^)	0.12	−1.4 × 10^−3^	(−4.6 × 10^−3^, 1.3 × 10^−3^)	0.31	−7.8 × 10^−4^	(−2.9 × 10^−3^, 4.4 × 10^−4^)	0.29	3.4 × 10^−4^	(−1.9 × 10^−3^, 3.1 × 10^−3^)	0.84

Note: cells are highlighted yellow when 0.05/14 ≤ *p* < 0.05. * Indicates that the original SNP from the UK Biobank analysis in Table 2 was not available, so another SNP was used. ** Full citations listed in References section.

## Data Availability

Data are publicly available for the COPDGene study (https://www.ncbi.nlm.nih.gov/projects/gap/cgi-bin/study.cgi?study_id=phs000179.v1.p1) and UK Biobank (https://www.ukbiobank.ac.uk/enable-your-research/apply-for-access).

## Supplementary Materials

The following supporting information can be downloaded at: https://www.mdpi.com/article/10.3390/genes15050565/s1. Table S1 and Table S2 contain the mediation analysis results similar to Table 2 and Table 3 but with the additional adjustment for age, sex, genetic ancestry via PCs, income, education, and location (urban vs. rural) in the UK biobank and COPDGene studies respectively. In Figures S1 and S2, plots depict the effects from the mediation analyses for the SNPs rs6424532 [*LOC105378800*] and rs10501696 [*GRM5*], respectively. In Figures S3 and S4, the plot depicts the interaction of the SNP rs6424532 [*LOC105378800*] and rs10501696 [*GRM5*] with sex and the log of pack-years on the probability of depression on the logit scale. The COPDGene study acknowledgements are also provided in the supplement.
