# Supplementary material for: Examining the Effect of Genes on Depression as Mediated by Smoking and Modified by Sex
Source: Genes (Basel). 2024 Apr 27;15(5):565. doi: 10.3390/genes15050565 (PMC11120779; doi:10.3390/genes15050565)
Supplement: Supplementary file 1 [file genes-15-00565-s001.zip › genes-2946681-supplementary.pdf]

Supplemental Materials: Examining the Effect of Genes on Depression as Mediated by Smoking and Modified by Sex

Kirsten Voorhies[1], Julian Hecker[2], Sanghun Lee[3], Georg Hahn[4], Dmitry Prokopenko[5], Merry-Lynn McDonald[6,7,8], Alexander C. Wu[9], Ann Wu[1], John E. Hokanson[10], Michael H. Cho[2], Christoph Lange[11], Karin F. Hoth[12], and Sharon M. Lutz[1,11]

[1] Department of Population Medicine, Harvard Pilgrim Health Care Institute, Boston, MA, USA
[2] Channing Division of Network Medicine, Brigham and Women's Hospital and Harvard Medical School, Boston, MA, USA
[3] Department of Medical Consilience, Division of Medicine, Graduate School, Dankook University, Yongin, South Korea
[4] Brigham and Women's Hospital, Division of Pharmacoepidemiology and Pharmacoeconomics, and Department of Medicine of Harvard Medical School, Boston, MA, USA
[5] Genetics and Aging Research Unit and the McCance Center for Brain Health, Department of Neurology, Massachusetts General Hospital, Boston, MA, USA
[6] Department of Epidemiology, School of Public Health, University of Alabama at Birmingham, Birmingham, AL, USA
[7] Division of Pulmonary, Allergy and Critical Care Medicine, Department of Medicine, University of Alabama at Birmingham, Birmingham, AL, USA
[8] Department of Genetics, University of Alabama at Birmingham, Birmingham, AL, USA
[9] Harvard College, Cambridge, MA, USA
[10] Department of Epidemiology, University of Colorado Anschutz Medical Campus, Aurora, CO, USA
[11] Department of Biostatistics, T.H. Chan School of Public Health, Harvard University, Boston, MA, USA
[12] Department of Psychiatry and Iowa Neuroscience Institute, University of Iowa, Iowa City, IA, USA

Supplemental Table S1. Mediation, sex stratified mediation, and sex moderated mediation results for the indirect effect of the SNP on depression through log of pack-years of cigarette smoking in the UK Biobank adjusting for age, sex, genetic ancestry via PCs, income, education, and location (urban vs rural).

Chr	Marker	Gene/ nearest gene	Position	Allele Freq.	Prev. smok. assoc. **	Indirect Effect (All)			Indirect Effect (Female)			Indirect Effect (Male)			Sex Moderated Indirect Effect		
						Beta	95% CI	P	Beta	95% CI	P	Beta	95% CI	P	Beta	95% CI	P
1	rs10127497	*SGIP1*	66584461	0.14	30	-1.4E-5	(-3.8E-4, 3.6E-4)	0.95	-3.9E-5	(-6.8E-4, 6.2E-4)	0.91	-1.5E-5	(-4.4E-4, 4.0E-4)	0.94	-6.9E-8	(-7.7E-4, 6.8E-4)	0.98
1	rs6699744	*LOC105378797*	72359461	0.61	-	2.6E-4	(1.1E-5, 5.3E-4)	0.05	3.3E-4	(-1.4E-4, 7.9E-4)	0.16	2.1E-4	(-8.2E-5, 5.1E-4)	0.17	-9.5E-5	(-6.9E-4, 4.0E-4)	0.69
1	rs6424532	*LOC105378800*	73198339	0.49	-	4.7E-4	(2.2E-4, 7.3E-4)	8.0E-4	5.9E-4	(1.5E-4, 1.1E-3)	8.0E-3	3.6E-4	(6.7E-5, 6.5E-4)	0.02	-2.0E-4	(-6.9E-4, 3.9E-4)	0.45
1	rs7548151	*ASTN1*	177057847	0.08	30, 31	3.4E-4	(-1.4E-4, 8.0E-4)	0.16	1.1E-4	(-7.2E-4, 9.2E-4)	0.76	4.8E-4	(-5.6E-5, 1.0E-3)	0.08	3.9E-4	(-3.8E-4, 1.3E-3)	0.42
5	rs40465	*LOC105379109*	104646025	0.33	-	-1.1E-4	(-3.9E-4, 1.6E-4)	0.40	-1.2E-4	(-6.0E-4, 3.5E-4)	0.64	-1.2E-4	(-4.4E-4, 2.0E-4)	0.46	1.6E-5	(-4.2E-4, 5.9E-4)	0.94
6	rs3132685	*HCG9*	29978172	0.13	-	3.7E-4	(-2.5E-5, 7.6E-4)	0.06	5.0E-4	(-1.6E-4, 1.2E-3)	0.14	2.4E-4	(-1.8E-4, 6.7E-4)	0.26	-2.8E-4	(-1.1E-3, 4.8E-4)	0.43
6	rs112348907	*KCNQ5*	72878230	0.30	30	-3.3E-5	(-3.2E-4, 2.4E-4)	0.82	6.6E-5	(-4.5E-4, 5.6E-4)	0.81	-7.8E-5	(-4.1E-4, 2.4E-4)	0.64	-1.3E-4	(-6.4E-4, 4.2E-4)	0.74
7	rs3807865	*TMEM106B*	12210776	0.41	-	1.6E-4	(-1.0E-4, 4.2E-4)	0.22	3.3E-4	(-1.4E-4, 8.0E-4)	0.16	5.3E-5	(-2.4E-4, 3.5E-4)	0.73	-2.8E-4	(-7.6E-4, 2.1E-4)	0.31
7	rs2402273	*LSM8 / CTTNBP2*	117960370	0.41	30, 32-39	1.5E-4	(-1.1E-4, 4.2E-4)	0.26	-1.3E-4	(-5.9E-4, 3.2E-4)	0.57	3.3E-4	(4.5E-5, 6.4E-4)	0.02	5.0E-4	(-6.6E-5, 9.7E-4)	0.08
9	rs263575	*BNC2 / CNTLN*	17033842	0.46	30, 31	-1.6E-4	(-4.3E-4, 9.7E-5)	0.22	-4.2E-4	(-8.8E-4, 2.9E-5)	0.07	-3.2E-6	(-2.9E-4, 2.9E-4)	0.96	4.1E-4	(-3.9E-5, 9.4E-4)	0.11
10	rs1021363	*SORCS3*	104851081	0.64	30	-2.0E-4	(-4.6E-4, 6.5E-5)	0.13	-2.5E-4	(-7.3E-4, 2.1E-4)	0.29	-1.6E-4	(-4.6E-4, 1.6E-4)	0.29	1.3E-4	(-4.2E-4, 7.3E-4)	0.73
11	rs10501696	*GRM5*	89014994	0.50	30, 40, 41	-3.0E-4	(-5.6E-4, -3.4E-5)	0.03	-6.4E-4	(-1.1E-3, -1.8E-4)	3.8E-3	-8.7E-5	(-3.9E-4, 2.2E-4)	0.59	5.6E-4	(-3.1E-5, 1.2E-3)	0.05
13	rs9530139	*B3GLCT*	31273187	0.19	30	2.3E-5	(-3.0E-4, 3.4E-4)	0.89	-2.5E-4	(-8.4E-4, 2.9E-4)	0.39	1.9E-4	(-1.9E-4, 5.7E-4)	0.31	4.4E-4	(-1.3E-4, 1.0E-3)	0.15
15	rs28541419	*MRPL46*	88402647	0.23	30	3.4E-5	(-2.6E-4, 3.4E-4)	0.85	-1.2E-4	(-6.5E-4, 4.2E-4)	0.65	1.0E-4	(-2.1E-4, 4.8E-4)	0.47	2.4E-4	(-4.2E-4, 8.6E-4)	0.75

Note: cells are highlighted green when p < 0.05/14, and cells are highlighted yellow when 0.05/14 <= p < 0.05.
** Full citations listed in References section of the main manuscript.

Supplemental Table S2. Mediation, sex stratified mediation, and sex moderated mediation results for the indirect effect of the SNP on depression through log of pack-years of cigarette smoking in COPDGene adjusting for age, sex, genetic ancestry via PCs, income, education, and location (urban vs rural).

Chr	Marker	Gene/ nearest gene	Position	Allele Freq.	Prev. smok. assoc. **	Indirect Effect (All)			Indirect Effect (Female)			Indirect Effect (Male)			Sex Moderated Indirect Effect		
						Beta	95% CI	P	Beta	95% CI	P	Beta	95% CI	P	Beta	95% CI	P
1	rs10127497	*SGIP1*	66584461	0.14	30	-8.2E-6	(-1.3E-3, 1.3E-3)	0.99	-3.5E-4	(-3.6E-3, 2.7E-3)	0.81	8.4E-5	(-9.3E-4, 1.3E-3)	0.91	3.1E-04	(-2.2E-3, 2.9E-3)	0.87
1	rs6699744	*LOC105378797*	72360489*	0.20	-	1.5E-4	(-9.6E-4, 1.3E-3)	0.77	-9.1E-4	(-4.0E-3, 1.4E-3)	0.47	2.6E-4	(-8.3E-4, 2.0E-3)	0.64	1.5E-03	(-5.3E-3, 4.6E-3)	0.19
1	rs6424532	*LOC105378800*	73200931*	0.47	-	2.6E-4	(-7.5E-4, 1.3E-3)	0.53	-4.8E-4	(-2.9E-3, 1.4E-3)	0.60	2.7E-4	(-6.3E-4, 1.6E-3)	0.58	8.6E-04	(-7.0E-4, 3.0E-3)	0.31
1	rs7548151	*ASTN1*	177057847	0.09	30, 31	-3.6E-4	(-2.1E-3, 1.2E-3)	0.63	-9.0E-4	(-5.4E-3, 3.0E-3)	0.65	-8.7E-5	(-1.7E-3, 1.2E-3)	0.83	4.3E-04	(-3.4E-3, 3.8E-3)	0.91
5	rs40465	*LOC105379109*	104646025	0.33	-	7.5E-4	(-5.7E-5, 2.1E-3)	0.09	2.3E-3	(1.2E-4, 5.5E-3)	0.03	6.3E-5	(-6.9E-4, 1.1E-3)	0.90	-1.6E-03	(-4.4E-3, 6.1E-4)	0.18
6	rs112348907	*KCNQ5*	72878230	0.29	30	8.1E-4	(-1.3E-4, 2.2E-3)	0.11	5.9E-4	(-1.5E-3, 3.1E-3)	0.55	5.2E-4	(-8.7E-4, 2.8E-3)	0.47	6.9E-04	(-1.6E-3, 3.2E-3)	0.46
7	rs3807865	*TMEM106B*	12210776	0.41	-	-3.5E-4	(-1.5E-3, 4.5E-4)	0.43	4.0E-4	(-1.6E-3, 2.8E-3)	0.67	-3.3E-4	(-1.9E-3, 7.1E-4)	0.55	-1.1E-03	(-3.7E-3, 9.0E-4)	0.30
7	rs2402273	*LSM8 / CTTNBP2*	117960370	0.42	30, 32-39	6.4E-4	(-1.5E-4, 1.9E-3)	0.13	6.0E-4	(-1.6E-3, 2.9E-3)	0.54	2.9E-4	(-5.8E-4, 1.8E-3)	0.56	3.1E-04	(-2.5E-3, 3.1E-3)	0.81
9	rs263575	*BNC2 / CNTLN*	17033842	0.45	30, 31	-2.1E-4	(-1.2E-3, 6.5E-4)	0.64	1.4E-4	(-2.1E-3, 2.2E-3)	0.93	-1.7E-4	(-1.4E-3, 5.9E-4)	0.72	-5.0E-04	(-2.3E-3, 1.1E-3)	0.64
10	rs1021363	*SORCS3*	105109590*	0.03	30	-2.3E-3	(-5.7E-3, 1.8E-4)	0.07	-4.0E-3	(-0.01, 8.1E-4)	0.11	-7.9E-4	(-4.8E-3, 2.1E-3)	0.59	7.8E-04	(-5.3E-3, 6.9E-3)	0.77
11	rs10501696	*GRM5*	88584239*	0.01	30, 40, 41	-7.0E-3	(-1.6E-2, -4.0E-4)	0.03	-0.02	(-0.04, 1.0E-3)	0.08	-1.8E-3	(-0.01, 4.1E-3)	0.59	8.4E-03	(-7.4E-3,0.03)	0.40
13	rs9530139	*B3GLCT*	31273187	0.19	30	-6.6E-4	(-2.1E-3, 4.2E-4)	0.24	-9.4E-4	(-3.8E-3, 1.7E-3)	0.45	-3.1E-4	(-2.3E-3, 7.7E-4)	0.59	-3.8E-06	(-2.8E-3, 3.3E-3)	0.92
15	rs28541419	*MRPL46*	88402647	0.24	30	-8.5E-4	(-2.3E-3, 2.0E-4)	0.12	-1.4E-3	(-4.5E-3, 7.9E-4)	0.22	-3.1E-4	(-2.0E-3, 6.7E-4)	0.60	3.4E-04	(-1.9E-3, 3.1E-3)	0.84

Note: cells are highlighted green when p < 0.05/14, and cells are highlighted yellow when 0.05/14 <= p < 0.05.

** Full citations listed in References section of the main manuscript.

Supplemental Figure S1. These plots depict the effects from the mediation analyses for the SNP rs6424532 [*LOC105378800*]. For the plots in the top row, we adjusted for age, sex (except when stratified by sex), smoking status, and the first 8 PCs. For the plots in the bottom row we also adjusted for college education, low income, and urban living. The plots in column 1 are for the full sample (females and males), the plots in column 2 are females only, and the plots in column 3 are males only.

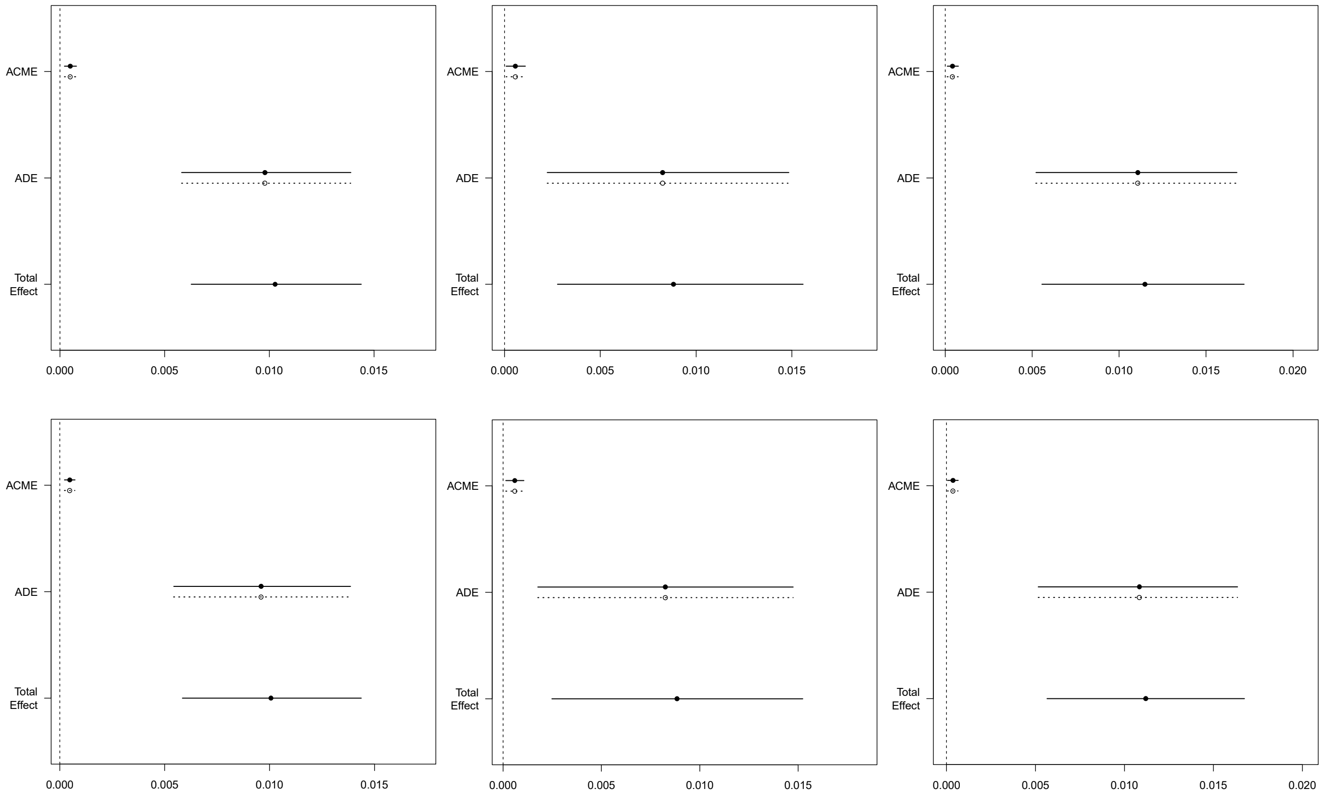

Supplemental Figure S2. These plots depict the effects from the mediation analyses for the SNP rs10501696 [*GRM5*]. For the plots in the top row, we adjusted for age, sex (except when stratified by sex), smoking status, and the first 8 PCs. For the plots in the bottom row we also adjusted for college education, low income, and urban living. The plots in column 1 are for the full sample (females and males), the plots in column 2 are females only, and the plots in column 3 are males only.

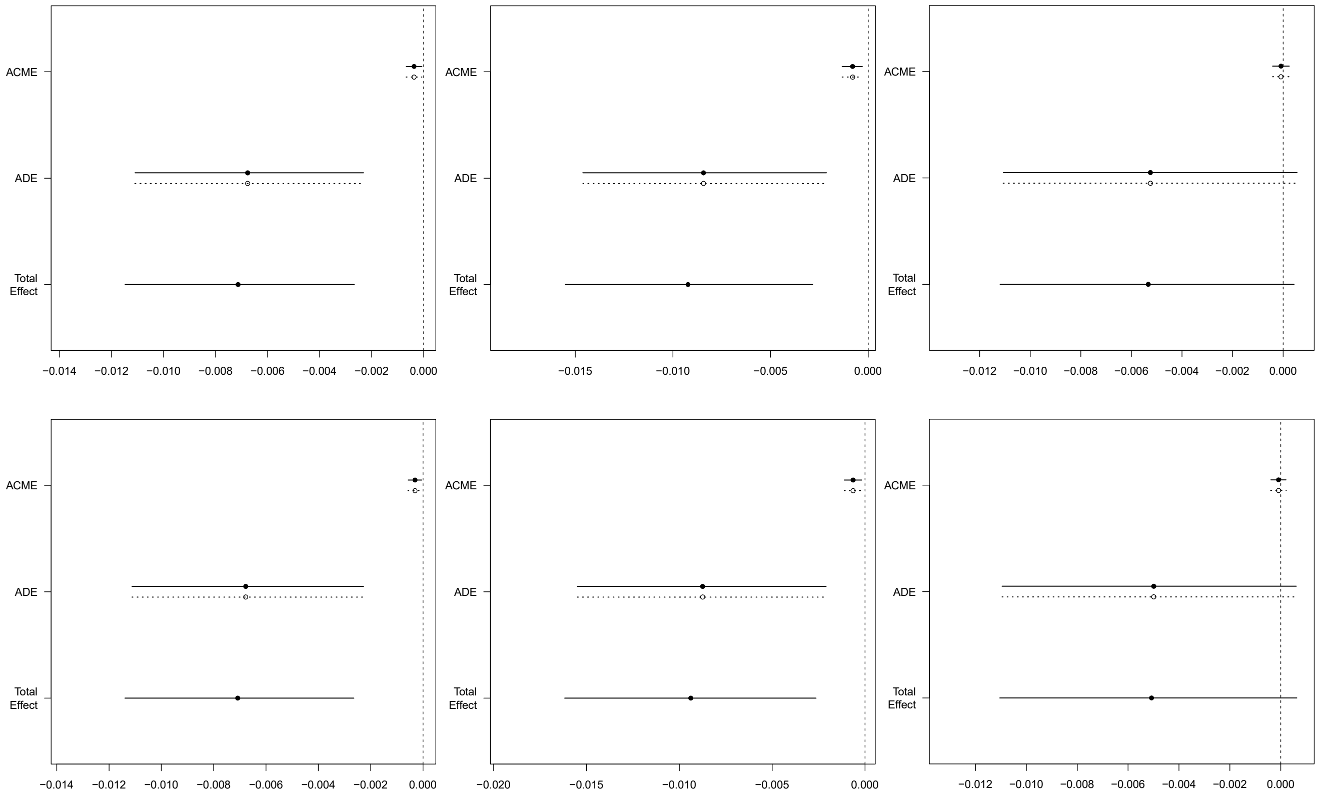

Supplemental Figure S3. The plot depicts the interaction of the SNP rs6424532 [*LOC105378800*] with sex and the log of pack-years on the probability of depression on the logit scale. For the legend, we define which colors correspond to female and which correspond to male with the corresponding number of risk alleles for the SNP rs6424532.

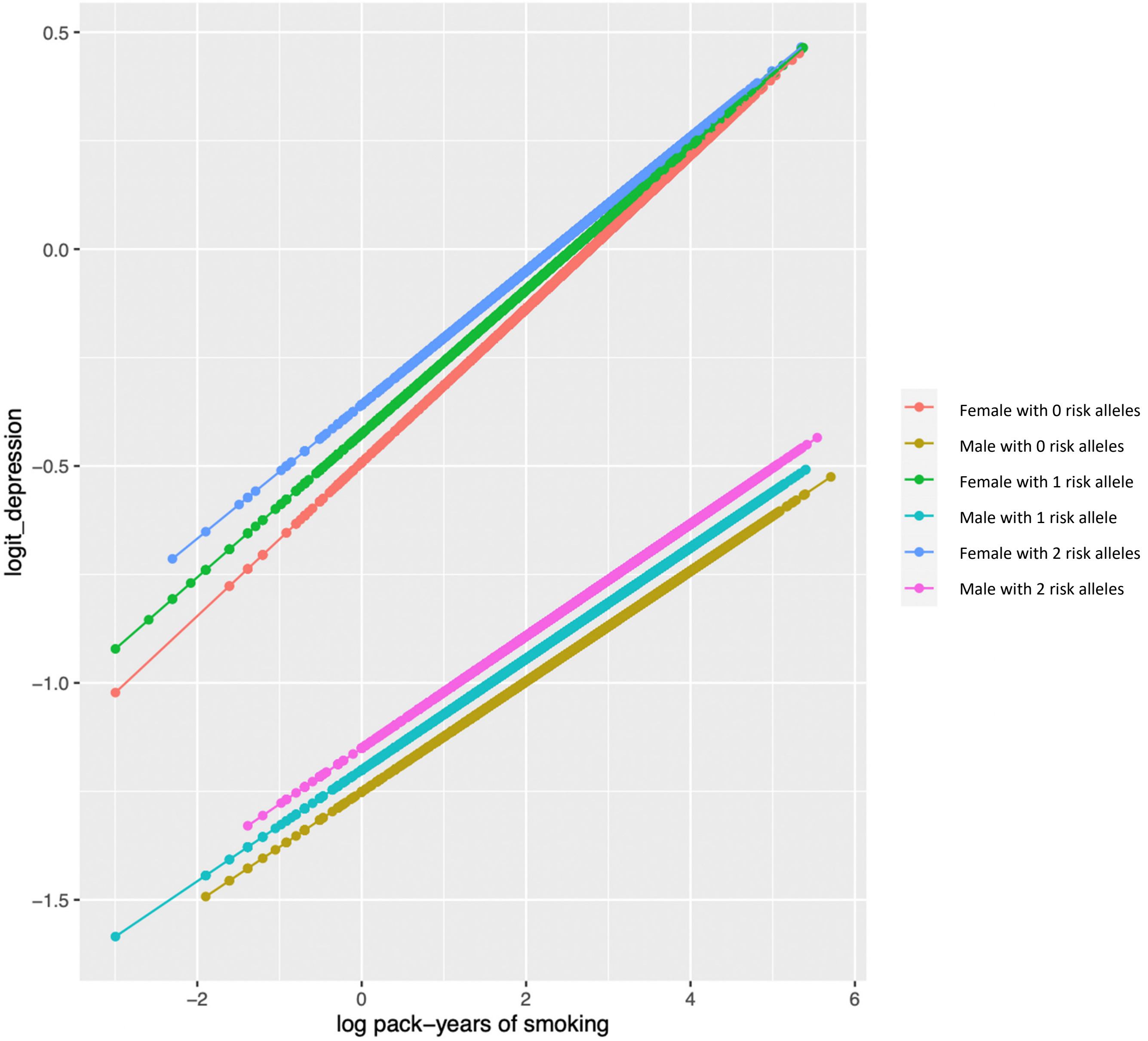

Supplemental Figure S4. The plot depicts the interaction of the SNP rs10501696 [*GRM5*] with sex and the log of pack-years on the probability of depression on the logit scale. For the legend, we define which colors correspond to female and which correspond to male with the corresponding number of risk alleles for the SNP rs10501696.

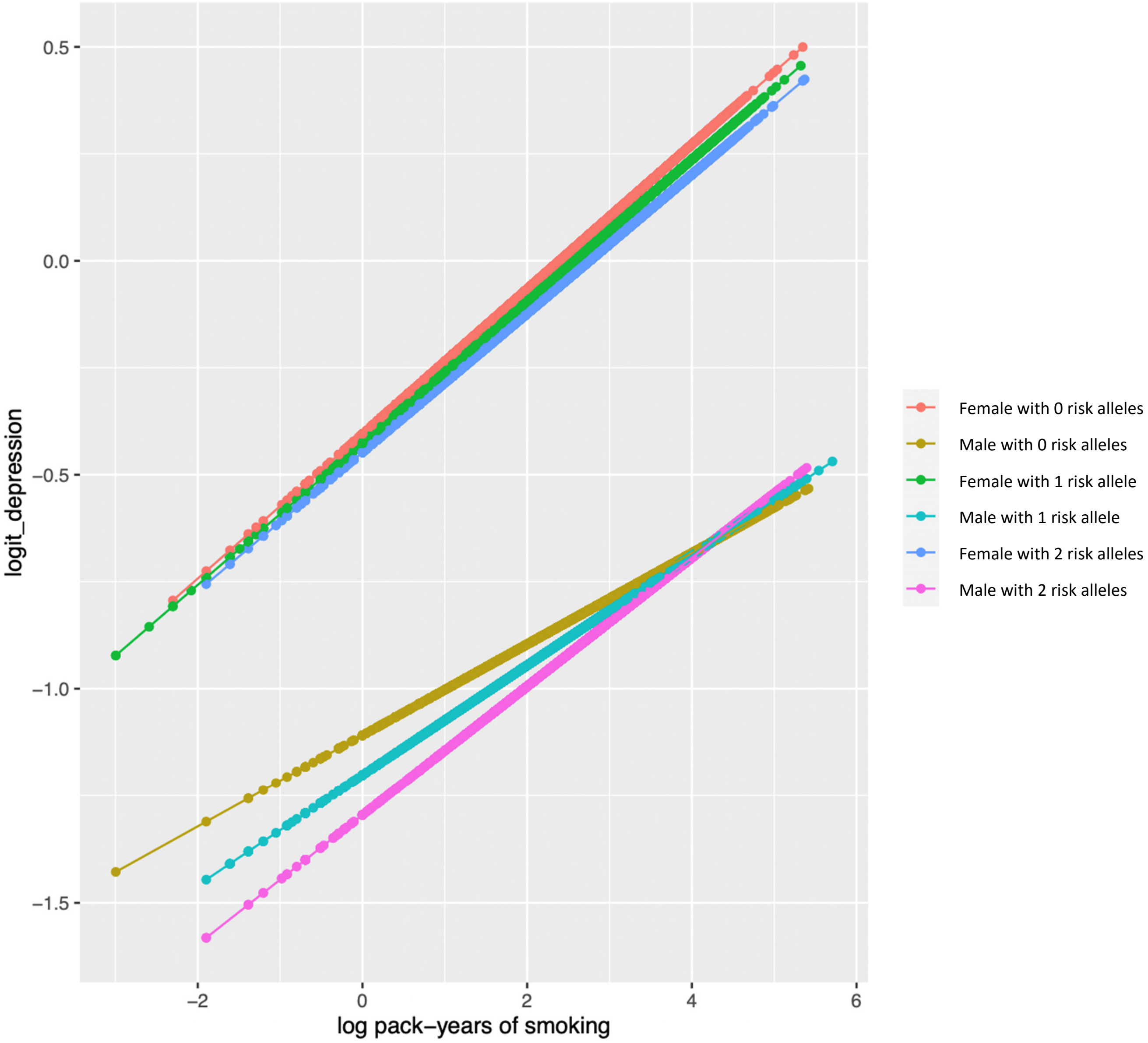

COPDGene Phase 3

Grant Support and Disclaimer

The project described was supported by Award Number U01 HL089897 and Award Number U01 HL089856 from the National Heart, Lung, and Blood Institute. The content is solely the responsibility of the authors and does not necessarily represent the official views of the National Heart, Lung, and Blood Institute or the National Institutes of Health.

COPD Foundation Funding

COPDGene is also supported by the COPD Foundation through contributions made to an Industry Advisory Board comprised of AstraZeneca, Boehringer-Ingelheim, Genentech, GlaxoSmithKline, Novartis, Pfizer, Siemens, and Sunovion.

COPDGene® Investigators – Core Units

Administrative Center: James D. Crapo, MD (PI); Edwin K. Silverman, MD, PhD (PI); Barry J. Make, MD; Elizabeth A. Regan, MD, PhD

Genetic Analysis Center: Terri Beaty, PhD; Ferdouse Begum, PhD; Peter J. Castaldi, MD, MSc; Michael Cho, MD; Dawn L. DeMeo, MD, MPH; Adel R. Boueiz, MD; Marilyn G. Foreman, MD, MS; Eitan Halper-Stromberg; Lystra P. Hayden, MD, MMSc; Craig P. Hersh, MD, MPH; Jacqueline Hetmanski, MS, MPH; Brian D. Hobbs, MD; John E. Hokanson, MPH, PhD; Nan Laird, PhD; Christoph Lange, PhD; Sharon M. Lutz, PhD; Merry-Lynn McDonald, PhD; Margaret M. Parker, PhD; Dmitry Prokopenko, Ph.D; Dandi Qiao, PhD; Elizabeth A. Regan, MD, PhD; Phuwanat Sakornsakolpat, MD; Edwin K. Silverman, MD, PhD; Emily S. Wan, MD; Sungho Won, PhD

Imaging Center: Juan Pablo Centeno; Jean-Paul Charbonnier, PhD; Harvey O. Coxson, PhD; Craig J. Galban, PhD; MeiLan K. Han, MD, MS; Eric A. Hoffman, Stephen Humphries, PhD; Francine L. Jacobson, MD, MPH; Philip F. Judy, PhD; Ella A. Kazerooni, MD; Alex Kluiber; David A. Lynch, MB; Pietro Nardelli, PhD; John D. Newell, Jr., MD; Aleena Notary; Andrea Oh, MD; Elizabeth A. Regan, MD, PhD; James C. Ross, PhD; Raul San Jose Estepar, PhD; Joyce Schroeder, MD; Jered Sieren; Berend C. Stoel, PhD; Juerg Tschirren, PhD; Edwin Van Beek, MD, PhD; Bram van Ginneken, PhD; Eva van Rikxoort, PhD; Gonzalo Vegas SanchezFerrero, PhD; Lucas Veitel; George R. Washko, MD; Carla G. Wilson, MS;

PFT QA Center, Salt Lake City, UT: Robert Jensen, PhD

Data Coordinating Center and Biostatistics, National Jewish Health, Denver, CO: Douglas Everett, PhD; Jim Crooks, PhD; Katherine Pratte, PhD; Matt Strand, PhD; Carla G. Wilson, MS

Epidemiology Core, University of Colorado Anschutz Medical Campus, Aurora, CO: John E. Hokanson, MPH, PhD; Gregory Kinney, MPH, PhD; Sharon M. Lutz, PhD; Kendra A. Young, PhD

Mortality Adjudication Core: Surya P. Bhatt, MD; Jessica Bon, MD; Alejandro A. Diaz, MD, MPH; MeiLan K. Han, MD, MS; Barry Make, MD; Susan Murray, ScD; Elizabeth Regan, MD; Xavier Soler, MD; Carla G. Wilson, MS

Biomarker Core: Russell P. Bowler, MD, PhD; Katerina Kechris, PhD; Farnoush BanaeiKashani, Ph.D

COPDGene® Investigators – Clinical Centers

Ann Arbor VA: Jeffrey L. Curtis, MD; Perry G. Pernicano, MD

Baylor College of Medicine, Houston, TX: Nicola Hanania, MD, MS; Mustafa Atik, MD; Aladin Boriek, PhD; Kalpatha Guntupalli, MD; Elizabeth Guy, MD; Amit Parulekar, MD;

Brigham and Women's Hospital, Boston, MA: Dawn L. DeMeo, MD, MPH; Alejandro A. Diaz, MD, MPH; Lystra P. Hayden, MD; Brian D. Hobbs, MD; Craig Hersh, MD, MPH; Francine L. Jacobson, MD, MPH; George Washko, MD

Columbia University, New York, NY: R. Graham Barr, MD, DrPH; John Austin, MD; Belinda D'Souza, MD; Byron Thomashow, MD

Duke University Medical Center, Durham, NC: Neil MacIntyre, Jr., MD; H. Page McAdams, MD; Lacey Washington, MD

Grady Memorial Hospital, Atlanta, GA: Eric Flenaugh, MD; Silanth Terpenning, MD

HealthPartners Research Institute, Minneapolis, MN: Charlene McEvoy, MD, MPH; Joseph Tashjian, MD

Johns Hopkins University, Baltimore, MD: Robert Wise, MD; Robert Brown, MD; Nadia N. Hansel, MD, MPH; Karen Horton, MD; Allison Lambert, MD, MHS; Nirupama Putcha, MD, MHS

Lundquist Institute for Biomedical Innovationat Harbor UCLA Medical Center, Torrance, CA: Richard Casaburi, PhD, MD; Alessandra Adami, PhD; Matthew Budoff, MD; Hans Fischer, MD; Janos Porszasz, MD, PhD; Harry Rossiter, PhD; William Stringer, MD

Michael E. DeBakey VAMC, Houston, TX: Amir Sharafkhaneh, MD, PhD; Charlie Lan, DO

Minneapolis VA: Christine Wendt, MD; Brian Bell, MD; Ken M. Kunisaki, MD, MS

National Jewish Health, Denver, CO: Russell Bowler, MD, PhD; David A. Lynch, MB

Reliant Medical Group, Worcester, MA: Richard Rosiello, MD; David Pace, MD

Temple University, Philadelphia, PA: Gerard Criner, MD; David Ciccolella, MD; Francis Cordova, MD; Chandra Dass, MD; Gilbert D'Alonzo, DO; Parag Desai, MD; Michael Jacobs, PharmD; Steven Kelsen, MD, PhD; Victor Kim, MD; A. James Mamary, MD; Nathaniel Marchetti, DO; Aditi Satti, MD; Kartik Shenoy, MD; Robert M. Steiner, MD; Alex Swift, MD; Irene Swift, MD; Maria Elena Vega-Sanchez, MD

University of Alabama, Birmingham, AL: Mark Dransfield, MD; William Bailey, MD; Surya P. Bhatt, MD; Anand Iyer, MD; Hrudaya Nath, MD; J. Michael Wells, MD

University of California, San Diego, CA: Douglas Conrad, MD; Xavier Soler, MD, PhD; Andrew Yen, MD

University of Iowa, Iowa City, IA: Alejandro P. Comellas, MD; Karin F. Hoth, PhD; John Newell, Jr., MD; Brad Thompson, MD

University of Michigan, Ann Arbor, MI: MeiLan K. Han, MD MS; Ella Kazerooni, MD MS; Wassim Labaki, MD MS; Craig Galban, PhD; Dharshan Vummidi, MD

University of Minnesota, Minneapolis, MN: Joanne Billings, MD; Abbie Begnaud, MD; Tadashi Allen, MD

University of Pittsburgh, Pittsburgh, PA: Frank Sciurba, MD; Jessica Bon, MD; Divay Chandra, MD, MSc; Carl Fuhrman, MD; Joel Weissfeld, MD, MPH

University of Texas Health, San Antonio, San Antonio, TX: Antonio Anzueto, MD; Sandra Adams, MD; Diego Maselli-Caceres, MD; Mario E. Ruiz, MD; Harjinder Singh
